# Delivering and Embedding Dementia-Friendly Training in a Healthcare System

**DOI:** 10.1093/geroni/igaa057.522

**Published:** 2020-12-16

**Authors:** Ellen Schneider, Maureen Dale, Krista Wells, John Gotelli, Carol Julian, Cristine Henage, Jan Busby-Whitehead, Ellen Roberts

**Affiliations:** 1 University of North Carolina at Chapel Hill, Raleigh, North Carolina, United States; 2 University of North Carolina School of Medicine, Chapel Hill, North Carolina, United States; 3 UNC Health Care, Hillsborough, North Carolina, United States

## Abstract

Alzheimer’s disease is the 4th leading cause of death in North Carolina for people 65 and older. People with dementia are hospitalized more often and have prolonged stays, poorer outcomes, higher costs, and increased readmission rates. Hospital employees have expressed the desire to have specialized training to learn how to more effectively communicate with and provide better care to patients with dementia. To address identified patient and hospital employee needs, the University of North Carolina (UNC) Center for Aging and Health is disseminating hospital-specific dementia-friendly training at five hospitals within the UNC Health System. The training is being delivered via online modules and follow-up didactic sessions over a three-year period to clinical and non-clinical staff who interact with patients. To date, 1,948 employees at three of the five hospitals have launched the online training; 1,102 have completed the training. The pilot training took place at the UNC Hospitals--Hillsborough Campus (“Hillsborough Hospital”) in 2019. Hillsborough Hospital staff (n=195) who participated in the dementia friendly training completed a survey to assess their ability to recognize symptoms and provide appropriate care to dementia patients pre- and post-training. Clinical staff answered 23 Likert scale self-efficacy questions; non-clinical staff answered the first 12 of these questions. Positive change in self-efficacy ratings from pre- to post-training was significant for every question (p < .0001). Additional results will be included in the poster. The dementia-friendly hospital initiative is preparing employees to provide better care for people with dementia and is effective in increasing employee self-efficacy.

